# Establishing a global medical physics graduate clinical training and development program in Ghana: A model for global health international education and collaboration

**DOI:** 10.1002/acm2.70410

**Published:** 2025-12-15

**Authors:** Shannon E. O'Reilly, Stephen Avery, Lyna Dinh, Andrew Friberg, Ayoola Okuribido, Eric K. Addison, Stephen Inkoom, Alhassan Mohammed Baidoo, Samuel Nii Tagoe, Elsie Effah Kaufmann, Sonya Gwak, Megan L. Doherty, Edem Sosu, Beatrice Wiafe Addai, Francis Hasford

**Affiliations:** ^1^ Department of Human Oncology, School of Medicine and Public Health University of Wisconsin Madison Wisconsin USA; ^2^ Department of Radiation Oncology, Perelman School of Medicine University of Pennsylvania Philadelphia Pennsylvania USA; ^3^ Department of Radiation Oncology UPMC Hillman Cancer Center Pittsburgh Pennsylvania USA; ^4^ Oncology Directorate Komfo Anokye Teaching Hospital Kumasi Ghana; ^5^ Ghana Atomic Energy Commission School of Nuclear and Allied Sciences University of Ghana Accra Ghana; ^6^ Department of Medical Physics Sweden Ghana Medical Centre Accra Ghana; ^7^ Department of Radiotherapy Korle Bu Teaching Hospital Accra Ghana; ^8^ School of Engineering Sciences University of Ghana Accra Ghana; ^9^ School of Engineering University of Pennsylvania Philadelphia Pennsylvania USA; ^10^ Center for Global Health, Perelman School of Medicine University of Pennsylvania Philadelphia Pennsylvania USA; ^11^ Peace and Love Hospital Kumasi Ghana

**Keywords:** collaboration, education, global

## Abstract

**Purpose:**

The Global Medical Physics Training and Development Program (GMPTDP) is a novel initiative that provides United States (US)‐based graduate students in medical physics with structured, immersive clinical training in Ghana.

**Methods:**

The five‐week program begins with a cultural and clinical orientation in the US, followed by 4 weeks of clinical rotations across leading Ghanaian medical institutions. During rotations, students gain experience with teletherapy (LINACs and cobalt‐60), brachytherapy, treatment planning, imaging, and more. Trainees participate in clinical activities, conduct collaborative projects, and engage in community outreach and cultural immersion. The program culminates in a symposium highlighting student experiences and future directions with speakers including physicists, oncologists, engineers, and policymakers.

**Results:**

The pilot year of the program was successfully completed by three students from May 28 2024–July 2 2024. This article outlines the development, structure, and implementation of GMPTDP as a replicable model for global health training in medical physics, emphasizing sustainable partnerships between high‐income and low‐ and middle‐income countries. Educational objectives include demonstrating effective cross‐border training models, fostering collaborative research, and expanding global clinical experience in the field of medical physics.

**Conclusions:**

A model for a global medical physics training program was developed and successfully implemented.

## 1 INTRODUCTION

The Global Medical Physics Training and Development Program (GMPTDP) was created to provide United States (US)‐based medical physics graduate students (MS and PhD) in a CAMPEP‐accredited program with a unique opportunity to enhance their clinical training through global engagement. It aims to introduce students to international career opportunities while strengthening collaborative ties between institutions in the United States and Africa. The program is grounded in the recognition that advancing medical physics globally, particularly in low‐ and middle‐income countries (LMICs), requires targeted education and capacity‐building efforts.

The program aligns with key international goals, including those outlined in the 2022 Lancet Oncology Commission report on cancer in sub‐Saharan Africa,^1^ which emphasized the urgent need to expand the oncology workforce. There is a global shortage of medical physicists^2^ and an estimated surge in radiotherapy workforce need of over 60% by 2050^3^ due to the increase in cancer burden. The GMPTDP also supports the United Nations Sustainable Development Goals (SDGs),^4^ particularly SDG 4 (ensuring quality education) and SDG 17 (global partnerships). Education and global partnerships are essential for capacity‐building efforts. An American Association of Physicists in Medicine (AAPM) survey of radiology and radiotherapy leaders in LMICs found that the greatest infrastructure need was training.^5^ They also reported that over 85% of respondents would support global collaborations.

Participants in the GMPTDP gain a deeper understanding of global health frameworks and develop practical skills by working in resource‐constrained environments. Trainees engage in hands‐on clinical experiences, research projects, and collaborative activities focused on the implementation of emerging technologies such as artificial intelligence (AI) tools in radiotherapy workflows. By rotating through multiple centers across Ghana, students develop a broader perspective on health system infrastructure, cancer care delivery, and the role of medical physics in global health. Students are expected to reach a greater understanding and appreciation of the impact that culture, society, politics, economics, and technologies have on improving health, education, and quality of life.

## 2 INNOVATION

The GMPTDP is the only global clinical training program of this nature at the medical physics graduate program level in the United States. This program is the result of an extensive, collaborative effort between three institutions and four medical centers in the US and Ghana, specifically: University of Pennsylvania (UPenn), University of Ghana (UG), Kwame Nkrumah University of Science and Technology (KNUST), Sweden Ghana Medical Centre (SGMC), Komfo Anokye Teaching Hospital (KATH), Korle Bu Teaching Hospital (KBTH), and Peace and Love Hospital (P&L). This collaboration has the potential to be consequential throughout the region, as Ghana has become a hub for medical physics training and research in sub‐Saharan Africa, as it is home to the only International Atomic Energy Agency (IAEA) Regional Designated Centre for Medical Physics Training in Africa.^6^

### 2.1 Program development

**FIGURE 1:**
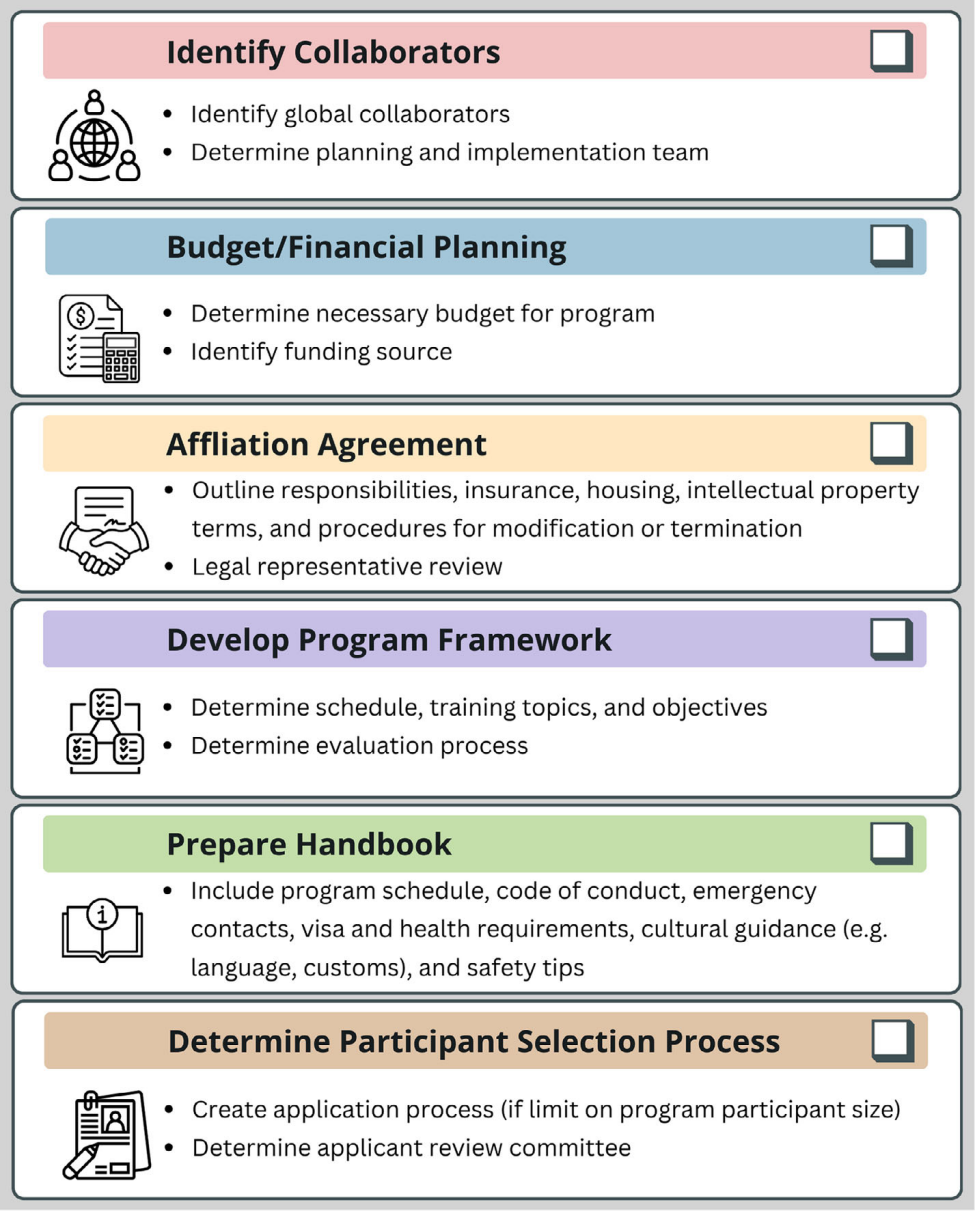
Generalized checklist for forming a new global training program.

Formal planning for the GMPTDP began in Fall 2022, initiated through collaborative meetings between colleagues in the US and Ghana and the submission of funding proposals. The program development team included physicists, a physician, engineering faculty, as well as UPenn's Director of Operations in Global Health, who has extensive experience developing peer learning programs with Ghanaian colleagues. Garnering departmental and institutional support was a key aspect of program development, as was establishing a budget. This program was built as a global extension of the clinical practicum course offered at UPenn, which allows graduate students to gain clinical experience at a US‐based site (main, network, or affiliate). A crucial element to the creation of such a program is building a global collaboration. International organizations such as the Global Health Catalyst, Medical Physics for World Benefit, the International Council of the AAPM, and the International Organization of Medical Physics provide a strong network for global connections. Figure 1 provides a generalized checklist for forming a new global training program.

In the Fall of 2023, faculty from the University of Pennsylvania traveled to Ghana (Accra and Kumasi) on a logistics and planning trip supported by Penn Global seed funding. During this visit, the team held strategic meetings with government representatives from the Ministry of Health, faculty from the University of Ghana, and staff from each participating medical center. Site tours and consultations with physicists and radiation oncologists were conducted to refine program logistics and confirm training capabilities. Housing options in both cities were also evaluated. The budget was determined based on prices in Ghana (housing, food, and local transportation) and included costs for travel medicine, visas, flights, and computational resources for AI tools.

To foster early collaboration and peer exchange, a peer‐to‐peer network was launched in March 2023 between the students at the University of Pennsylvania and the University of Ghana. This platform included virtual group meetings, chat‐based discussions, and matched partnerships based on shared academic and clinical interests. A survey was used to inform pairings and guide topical discussions. This survey included questions on medical physics track interests (diagnostic, therapy, and/or nuclear medicine), research interests, and preferred methods of communication.

Importantly, this peer‐to‐peer initiative provided preliminary data supporting the NIH R25 *AMPERE* (Access for Medical Physicists to Education and Research Excellence) program, which is currently under negotiation for funding. A distinguishing feature of this proposal is its emphasis on leveraging immersive technologies, particularly AI‐driven collaboration tools and low‐cost virtual reality (VR) platforms, to overcome geographic and resource barriers. By piloting structured peer engagement in advance, the GMPTDP positioned students to transition seamlessly into these innovative environments. Within this framework, AI/VR integration is not an ancillary component but a deliberate strategy to ensure that US graduate students and their LMIC peers gain early exposure to emerging tools that are already reshaping radiation oncology practice (e.g., AI‐assisted treatment planning and VR‐enabled training simulations). This linkage strengthens the educational infrastructure, aligns with the broader goals of the GMPTDP, and underscores the program's focus on AI/VR integration as both timely and essential to preparing the next generation of medical physicists for global, technology‐enabled clinical practice.

A formal affiliation agreement between partner institutions was established prior to the summer program launch. This agreement outlined responsibilities, insurance, housing, intellectual property terms, and procedures for modification or termination. Legal and academic representatives from each institution reviewed and approved the agreement. Furthermore, the affiliation agreement was written to be open to future opportunities for Ghanaian students to also come to the US. Additionally, a comprehensive student handbook was distributed prior to departure. It included a detailed program schedule, code of conduct, emergency contacts, visa and health requirements, packing checklist, and cultural guidance covering Ghanaian history, language, customs, and safety tips.

For the pilot year, the program supported three students (two MS and one PhD), although funding would allow for up to five annually for the subsequent two years. The program was open to all MS and PhD students in the US‐based graduate program who were taking the clinical practicum course that summer. These students had completed at least two semesters of CAMPEP courses and had some prior clinical training in patient‐specific quality assurance. Due to strong interest, a competitive application process was instituted. Applicants submitted a statement of interest addressing relevant international or LMIC experience, anticipated benefits of participation, and any background in AI or coding, given the program's focus on AI integration in clinical practice. The program committee selected participants based on these submissions. Of the 10 students taking the clinical practicum course that summer, seven applied for the global option.

### 2.2 Program schedule

The training program lasted five weeks and mirrored the required clinical practicum course offered to University of Pennsylvania graduate students while also incorporating training topics specific to global health. As a result, students enrolled in the UPenn CAMPEP‐accredited graduate programs were able to count this international rotation toward their clinical training requirements for graduation. The first week consisted of an orientation in the US, and the remaining four weeks involved rotations through four medical centers in Ghana.

#### 2.2.1 Orientation

A week‐long orientation was held prior to students traveling to Ghana. Speakers included directors from the UPenn Center for Global Health, a public health scholar from KNUST, physicists from Ghana, and specialists in AI and engineering. An overview of Ghanaian history, burden of disease in Ghana, health and safety tips, clinical professionalism, and planned cultural outings were discussed. Students were virtually introduced to physicists in Ghana who provided a clinical overview of training sites and equipment. Requirements of the program, presentation expectations, and possible clinical projects were also reviewed.

#### 2.2.2 Clinical rotations

After the pre‐departure orientation in the US, students trained at four clinical sites across Ghana: the Sweden Ghana Medical Centre, Korle Bu Teaching Hospital, Komfo Anokye Teaching Hospital, and Peace and Love Hospital. Each site exposed students to a range of radiotherapy modalities and imaging technologies, including linear accelerators (LINAC), cobalt‐60 machines, brachytherapy systems (HDR and LDR), simulators, computed tomography (CT), magnetic resonance imaging (MRI), mammography, and ultrasound. Students gained hands‐on experience with 3D conformal radiotherapy (3DCRT), intensity‐modulated radiotherapy (IMRT), quality assurance (QA), and treatment planning. Additional emphasis was placed on contextual factors such as equipment variability, infrastructure limitations, and clinical workflow in a resource‐limited setting. The role of a private versus public institution in cancer management was also examined.

**FIGURE 2:**
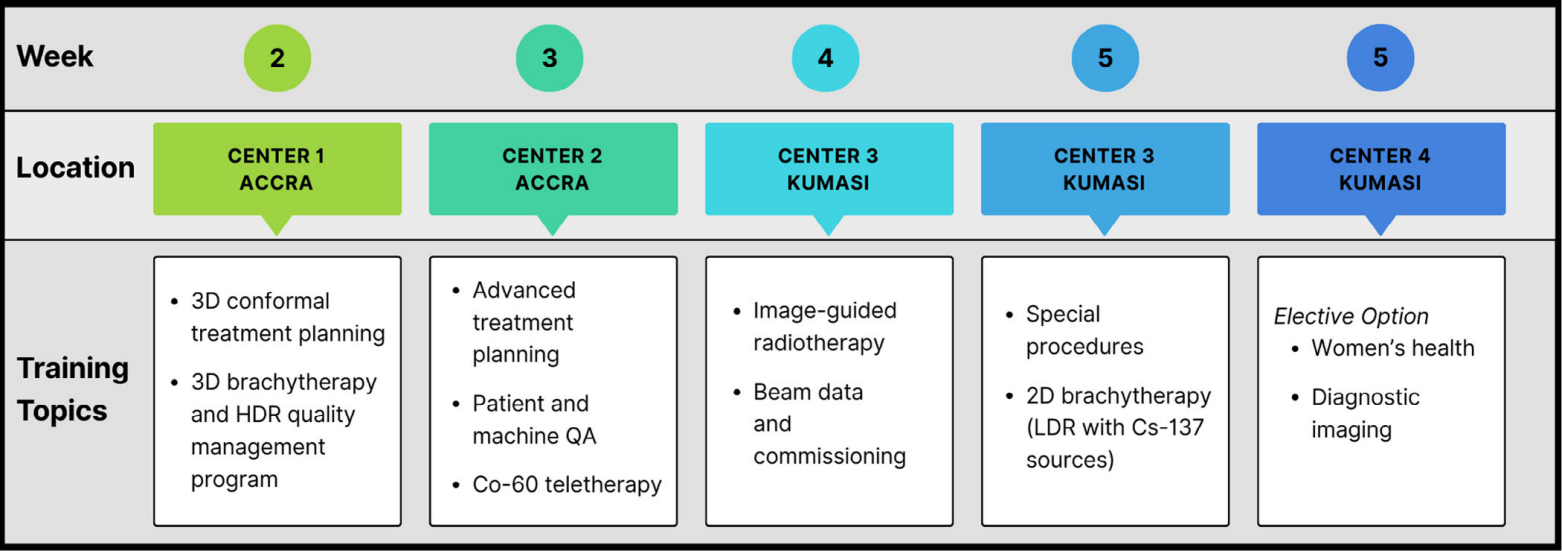
Weekly program training schedule.

The rotation schedule included defined weekly objectives aligned with clinical competencies and supported by references to AAPM Task Group reports, ICRU guidelines, and ESTRO booklets. Notably, students who elected the women's health track had an opportunity to engage in diagnostic imaging and community‐based breast cancer outreach at Peace and Love Hospital. During the pilot year, all students participated in this elective track. Figure 2 provides a weekly breakdown of what training topics were covered at each center.

#### 2.2.3 Clinical projects and student evaluation

Each student participated in site‐specific clinical projects designed to reinforce technical training, build upon didactic training, foster problem‐solving, and promote analytical thinking. These project results were formally presented to physicists and physicians at each center, enhancing students’ scientific communication skills. The students also wrote a project report for their first clinical project. Additionally, clinical workflows were examined to identify possible points where future AI implementation may be beneficial. Mid‐ and end‐of‐semester evaluation forms were completed for each student for the clinical practicum. The evaluation criteria included professionalism, clinical knowledge, and practical skills, with an evaluation scale of excellent, good, fair, or poor.

At the Sweden Ghana Medical Centre, students completed a 3D treatment planning assignment. After initial training by local physicists, students independently created plans for assigned treatment sites. They each analyzed a cohort of 20 patient cases, evaluating plan quality based on standard dosimetry metrics.

At the Korle Bu Teaching Hospital, students compared prostate treatment plans using cobalt‐60 and LINAC‐based approaches, considering the possibility of switching between machines in cases of limited availability. For each modality, they created both four‐field boxes and six‐field plans. They then compared the homogeneity and conformity indices, treatment times, and organ‐at‐risk constraints.

At the Komfo Anokye Teaching Hospital, students created and evaluated IMRT and 3DCRT plans for prostate, cervical, and head‐and‐neck cases. Across 16 plans (10 prostate, four cervical, two head‐and‐neck), they assessed planning target volume coverage, dose homogeneity, conformity, and normal tissue sparing. Additionally, students developed a prototype online QA tracking system using Python and Flask to digitize linear accelerator QA records and streamline documentation workflows.

#### 2.2.4 Outreach activities and cultural engagement

Beyond clinical work, the GMPTDP emphasized cultural immersion and community engagement to better appreciate the Ghanaian context. Students participated in multiple cultural outings and health education activities alongside Ghanaian peers and faculty. These activities enriched students’ understanding of Ghanaian society and history, while fostering cross‐cultural appreciation and respect.

A key outreach experience took place while rotating at the Peace and Love Hospital, where students joined a breast cancer screening awareness initiative at a local high school. They also had the opportunity to be part of a celebration with survivors hosted by Breast Care International (BCI), which highlighted the role of advocacy and education in promoting early detection and reducing stigma.

Cultural excursions included visits to historical and heritage sites such as the Elmina Castle, Bonwire Kente Weaving Village, Kwame Nkrumah Memorial Park, and the Manhyia Palace Museum, offering students insights into Ghana's political, artistic, and social history. Students also visited Kakum National Park, where they completed a canopy walk through the rainforest, and explored the Boabeng Fiema Monkey Sanctuary, a community‐managed conservation area. Additionally, trainees were able to try a plethora of dishes representing different regions of Ghana throughout the experience.

#### 2.2.5 End of program symposium

The program concluded with a full‐day symposium in Accra, coordinated with the Global Health Catalyst initiative. The event showcased the clinical, educational, and cultural experiences of GMPTDP trainees and served as a platform to engage policymakers, academic leaders, clinicians, and researchers from both the US and Ghana.

Student presentations highlighted key takeaways from their clinical rotations and collaborative projects, emphasizing lessons in global health, systems‐based practice, and intercultural learning. Sessions featured perspectives from Ghana's Ministry of Health, the Ghana Atomic Energy Commission, academic leaders, and international collaborators from institutions such as MD Anderson and the University of Washington.

Key themes of the symposium included:

1. Cancer control and workforce development in Ghana
2. Training and certification policies for allied health professionals
3. AI integration in radiation oncology
4. Proposed centers of excellence in AI, biomedical engineering, and global oncology research

The event reinforced the importance of long‐term, bilateral partnerships and provided a venue to generate future project ideas, including joint research initiatives and expanded educational opportunities.

#### 2.2.6 Student feedback

Upon completion of the summer training program, the three participating students were interviewed about their experience.^7^ Students emphasized the global perspectives gained:

Student 1: “*In Ghana, I learned a lot from a global perspective that I don't think I would have gotten anywhere else*.”

Student 2: “*My biggest takeaway from the experience was the need to take a holistic approach to cancer care. I now understand the public health initiative and how pushes for periodic diagnostic or screening exams can greatly contribute to reductions in cancer rates*.”

Student 3: “*Eventually, I'd like to work in lower‐middle income countries like Vietnam and Ghana*.”

They described developing a greater understanding of the challenges in working in resource‐constrained environments:

Student 1: “*It was a privilege to observe Ghanaian physicists across multiple hospitals, including their use of Cobalt‐60 machines. Despite challenges, the dedication of the physicists and their collaborations with engineering departments to solve issues and continue to provide patient care was inspiring*.”

Student 3: “*Sometimes you don't have access to the resources like we have at Penn. The Ghanaians taught me to be more resourceful*.”

The students discussed the clinical experience they gained:

Student 2: “*Over five intense weeks, the program allowed me to gain some hands‐on treatment planning experience on different machines, software systems, and modalities.” “We were able to observe some of the HDR procedures and even perform supervised brachytherapy afterloader and Linac quality assurance*.”

Student 3: “*Since Cobalt‐60 machines are not as common in the U.S., this was a novel opportunity for me to learn about teletherapy and the considerations involved*.”

One student also described the reciprocal nature of learning within the program:

Student 3: “*They were always willing to answer our questions, even if they were busy. And they were very open to learning from us as well. We were learning from each other*.”

Furthermore, students emphasized gaining a deeper understanding of medical physics:

Student 1: “*The trip really grounded me in why the field of medical physics is important and why we do what we do, why we study, why we research, why we advocate. It was the trip of a lifetime*.”

Student 2: “*And the knowledge we gained really helped put much of the things we learned in school into perspective*.”

#### 2.2.7 Workshop: Empowering future leaders in cancer care

Due to travel restrictions in 2025, international students enrolled at UPenn were unable to participate in the planned GMPTDP clinical rotations. To maintain program momentum and ensure continuity, a landmark three‐day workshop was held from July 2 2025 to July 4 2025 at KNUST in Kumasi, Ghana. Titled “Empowering Future Leaders in Cancer Care: VR & AI for Transforming Cancer Care in LMICs,” the workshop brought together oncologists, medical physicists, radiation therapists, and students to explore cutting‐edge strategies for implementing AI and virtual reality tools in clinical training and cancer treatment workflows. This three‐day event was aligned with the GMPTDP's objectives of strengthening research, clinical, and educational infrastructure, with a focus on AI, VR, and leadership training. Although the participants were not the same cohort as the initial GMPTDP students, one UPenn student, unaffected by travel restrictions, contributed as a facilitator. This reinforced program continuity and prepared the ground for student rotations to resume in 2026. These programmatic adaptations illustrate the flexibility of the GMPTDP model and its emphasis on sustaining partnerships even when global challenges arise.

The workshop featured keynote talks, virtual demonstrations, group exercises, and leadership panel discussions. Notable sessions included a presentation on the HypoAfrica clinical trial,^8^ live demonstrations of RadFormation and MD Anderson‐developed Radiation Planning Assistant tools,^9^ and hands‐on VR training modules developed in collaboration with the IAEA. Each Ghanaian clinical site, including KBTH, KATH, SGMC, and P&L, designed site‐specific AI workflows for clinical use.

Participants also engaged in breakout groups to refine AI integration strategies and completed a train‐the‐trainer session aimed at scaling education through local leadership. A highlight of the workshop was the leadership panel discussions featuring distinguished leaders in global oncology who shared insights from their journeys in advancing cancer care and biomedical education across Africa.

The workshop culminated in the identification of action items and next steps to sustain cross‐institutional partnerships, enhance education, and scale implementation of AI and VR‐based tools for cancer care in Ghana and beyond. It reinforced the GMPTDP's strategic vision of empowering a new generation of global health leaders trained in both clinical excellence and digital innovation.

## 3 DISCUSSION

### 3.1 Program review

The GMPTDP represents an adaptable and replicable model for international academic collaboration in medical physics. By integrating clinical education with cultural immersion and research, the program promotes mutual learning, intercultural responsiveness, and long‐term partnerships between institutions in high‐ and low‐resource settings. Trainees gained not only technical skills but also a nuanced understanding of the sociocultural, infrastructural, and economic factors influencing cancer care delivery in Ghana. The immersive nature of the program helped students appreciate the complexities of resource‐constrained environments and the critical role of local innovation in overcoming barriers to care. Additionally, students had a multifaceted medical physics experience as they worked not only with radiotherapy devices but also gained experience with diagnostic imaging. They also had the opportunity to work closely with physicians as the women's health rotation was led by a physician, clinicians attended their clinical project presentations, and they were involved in their brachytherapy training. Furthermore, they had the opportunity to attend clinical tumor boards, chart rounds, and shadow special procedures such as a lumpectomy and mastectomy.

Students also witnessed the challenges associated with equipment maintenance and limited diagnostic infrastructure. For example, one hospital's CT simulator was nonfunctional, requiring patients to be scanned at a private clinic across the street, incurring added cost and potential delays. These insights emphasized the need for sustainable engineering solutions and reliable service contracts as part of radiotherapy program development in LMICs.

Another critical observation was the persistent stigma surrounding cancer, particularly breast cancer. At Peace and Love Hospital, students heard firsthand accounts from survivors who shared how fear of abandonment, delayed diagnosis, and cultural misconceptions affected their treatment journey. This stigma can result in patients presenting with later‐stage disease, which the students observed during their clinical shadowing. The advocacy efforts of organizations like Breast Care International demonstrated the power of education and community outreach in shifting narratives and improving health‐seeking behavior. Through this experience, students not only contributed to clinical projects but also developed a broader worldview that will inform their future roles as global collaborators, educators, and leaders in medical physics.

The immersive nature of the GMPTDP has demonstrated the value of reciprocity, cultural exchange, and joint problem‐solving between US and Ghanaian partners. Beyond technical training, the program fosters a deeper understanding of systemic barriers to cancer care in LMICs and highlights the importance of sustainable collaboration. Preserving clinical productivity in LMIC facilities where staff resources are often limited is of utmost importance. In the context of this program, the integration of visiting students did not result in additional workload burdens for the host institutions. The educational model was primarily observational, with students engaging in structured shadowing of medical physicists and clinical staff during routine workflows. This approach minimized disruption, as staff did not have to suspend or significantly modify their activities for teaching purposes. Moreover, the students contributed in a manner similar to short‐term interns. Under supervision, they assisted with basic tasks and routine activities that, in their absence, would otherwise have been performed by full‐time staff. This dynamic created an element of workforce augmentation, where the presence of students provided ancillary support rather than diversion of resources. Accordingly, no additional full‐time equivalents (FTEs) were required for the training component of this initiative. Staff were able to integrate the trainees into ongoing operations without compromising service delivery. On the contrary, the collaboration was mutually beneficial: students gained clinical exposure within a resource‐limited setting, while staff experienced improved workflow efficiency and opportunities for professional exchange.

The program's emphasis on AI and VR integration reflects both the needs of the Ghanaian clinical context and the educational objectives of US graduate students. In Ghana, radiation oncology centers face workforce shortages, high patient volumes, and infrastructure variability. These conditions make the application of AI tools, such as automated treatment planning and workflow optimization, highly relevant for improving efficiency, reducing variability, and expanding access to safe radiotherapy. At the same time, VR‐based training modules, developed at the IAEA, provide scalable clinical simulations that are particularly valuable in resource‐limited environments where hands‐on access to advanced technologies may be constrained.

For US graduate students, engaging with AI/VR applications in Ghana offers dual benefits: exposure to innovations shaping the future of clinical medical physics and experience in adapting these technologies for diverse health systems. This dual perspective prepares students to be technically proficient and to lead the translation of emerging technologies into practice across settings. Thus, the AI/VR focus is well placed for the student population and aligned with the program's bilateral goals of strengthening education, research, and clinical capacity in both the US and Ghana.

The 2025 workshop exemplified this adaptability. Even when international rotations were not feasible, the program continued to deliver on its mission by convening Ghanaian clinicians, trainees, and future medical physics leaders for immersive training. This ensured that the program did not lose ground but rather expanded its reach and sustainability by cultivating local leadership. The integration of US student facilitation also demonstrated how the GMPTDP fosters a reciprocal, resilient, and scalable training model that can adapt to global challenges while maintaining its long‐term objectives. Looking ahead, this approach positions the GMPTDP to remain a model of innovation in global oncology education and a platform for advancing AI/VR‐driven radiotherapy training worldwide.

### 3.2 Future initiatives and collaborations

A key objective of the GMPTDP is to establish a lasting research and education partnership between the University of Pennsylvania and the University of Ghana, with an emphasis on AI implementation in radiotherapy. During the pilot year, workflow assessments and equipment audits were conducted to identify integration points for AI/VR tools, including applications in contouring, treatment planning, and quality assurance. Several AI developers have already committed to donating tools for non‐commercial use in LMICs. Future projects will evaluate these technologies in clinical settings and explore their use as teaching aids for trainees and practitioners. These collaborations may also evolve into graduate thesis projects, joint publications, and long‐term inter‐institutional research grants, which would enhance internationalization of the program.

To expand the program reach and impact, future plans include:

1. Extending the program duration and participant capacity beyond the current three‐year funding cycle.
2. Introducing a summer research fellowship track for undergraduate engineering students to strengthen the pipeline into medical physics and biomedical engineering graduate programs.
3. Exploring funding from the National Institutes of Health, ARPA‐H, and international organizations to support infrastructure development, curriculum exchange, and AI/VR research in radiation oncology.

By building upon the successes of the inaugural year, the GMPTDP aims to foster a robust academic infrastructure for global oncology training centered on reciprocity, innovation, and capacity building. This novel initiative aims at enhancing effective cross‐border training models, fostering collaborative research, and expanding global clinical experience in the field of medical physics, and supports the United Nations Sustainable Development Goals for quality education (SDG 4) and global partnerships (SDG 17).

## AUTHOR CONTRIBUTION

*Program development*: Stephen Avery, Shannon E. O'Reilly, Eric K. Addison, Stephen Inkoom, Samuel Nii Tagoe, Alhassan Mohammed Baidoo, Sonya Gwak, Megan L. Doherty, Elsie Effah Kaufmann, Edem Sosu, Beatrice Wiafe Addai, and Francis Hasford. *Writing of the manuscript*: Shannon E. O'Reilly, Stephen Avery, Lyna Dinh, Andrew Friberg, Ayoola Okuribido, and Megan L. Doherty. All authors contributed to the final review and revision of the manuscript as well as final approval of submission.

## CONFLICT OF INTEREST STATEMENT

The authors declare no conflicts of interest.

## Data Availability

Data sharing not applicable—no new data generated.
